# Response of Chloroplast NAD(P)H Dehydrogenase-Mediated Cyclic Electron Flow to a Shortage or Lack in Ferredoxin-Quinone Oxidoreductase-Dependent Pathway in Rice Following Short-Term Heat Stress

**DOI:** 10.3389/fpls.2016.00383

**Published:** 2016-03-30

**Authors:** Jemaa Essemine, Mingnan Qu, Hualing Mi, Xin-Guang Zhu

**Affiliations:** ^1^CAS Key Laboratory of Computational Biology, CAS-MPG Partner Institute for Computational Biology, Shanghai Institute for Biological Sciences, Chinese Academy of SciencesShanghai, China; ^2^National Key Laboratory of Plant Molecular Genetics, Shanghai Institute of Plant Physiology and Ecology, Chinese Academy of SciencesShanghai, China

**Keywords:** cyclic electron flow, FQR and NDH, heat stress, hcef and lcef, photosynthesis, P_700_, rice

## Abstract

Cyclic electron flow (CEF) around photosystem I (PSI) can protect photosynthetic electron carriers under conditions of stromal over-reduction. The goal of the research reported in this paper was to investigate the responses of both PSI and photosystem II (PSII) to a short-term heat stress in two rice lines with different capacities of cyclic electron transfer, i.e., Q4149 with a high capacity (hcef) and C4023 with a low capacity (lcef). The absorbance change at 820 nm (ΔA_820_) was used here to assess the charge separation in the PSI reaction center (P_700_). The results obtained show that short-term heat stress abolishes the ferredoxin-quinone oxidoreductase (FQR)-dependent CEF in rice and accelerates the initial rate of P_700_^+^ re-reduction. The P_700_^+^ amplitude was slightly increased at a moderate heat-stress (35°C) because of a partial restriction of FQR but it was decreased following high heat-stress (42°C). Assessment of PSI and PSII activities shows that PSI is more susceptible to heat stress than PSII. Under high temperature, FQR-dependent CEF was completely removed and NDH-dependent CEF was up-regulated and strengthened to a higher extent in C4023 than in Q4149. Specifically, under normal growth temperature, hcef (Q4149) was characterized by higher FQR- and chloroplast NAD(P)H dehydrogenase (NDH)-dependent CEF rates than lcef (C4023). Following thermal stress, the activation of NDH-pathway was 130 and 10% for C4023 and Q4149, respectively. Thus, the NDH-dependent CEF may constitute the second layer of plant protection and defense against heat stress after the main route, i.e., FQR-dependent CEF, reaches its capacity. We discuss the possibility that under high heat stress, the NDH pathway serves as a safety valve to dissipate excess energy by cyclic photophosphorylation and overcome the stroma over-reduction following inhibition of CO_2_ assimilation and any shortage or lack in the FQR pathway. The potential role of the NDH-dependent pathway during the evolution of C_4_ photosynthesis is briefly discussed.

## Introduction

Light energy is captured by plants through the LHCII and LHCI, which is then converted into chemical energy by the function of the two photosystems I and II (PSI and PSII). The two photosystems operate in tandem to drive the LEF to reduce NADP^+^, thereby forming the reducing power in the forms of reduced ferredoxin or NADPH in the stroma. Concomitantly, the e^-^ transport through the Cytb_6_/f, an intermediate complex between the two photosystems, generates a proton gradient across the thylakoid membrane (ΔpH) that is subsequently used by the ATP-synthase pump to synthesize ATP. Besides the LEF, ATP can be produced from CEF around PSI; therein electrons are recycled from reduced ferredoxin or NADPH to the PQ pool operating in the e^-^ transport from the PSII to the Cytb_6_/f (Bendall and Manasse, 1995). ATP and NADPH produced during the light reaction are mainly used by the Calvin–Benson and the photorespiration cycles. The CEF depends solely on the PSI photochemical reaction. CEF can generate a proton gradient (ΔpH) and drives ATP synthesis by ATP synthase without simultaneous generation of NADPH (Heber et al., 1978; Heber and Walker, 1992).

According to Shikanai’s (2007) review, two alternative pathways have been demonstrated for PSI-CEF in higher plants. The main pathway is mediated by the FQR, and two proteins, i.e., PGR5 (Munekage et al., 2002) and PGR5-LIKE1 (PGRL1; DalCorso et al., 2008). The second pathway is mediated by the NDH complex, a homolog of mitochondrial complex I (Mi et al., 1995; Burrows et al., 1998; Kofer et al., 1998; Shikanai et al., 1998). In *Arabidopsis thaliana*, research using mutants in which cyclic pathways were impaired showed that CEF is essential for an efficient photosynthesis and optimal growth (Munekage et al., 2004). Horváth et al. (2000) found that ndhB-deficient tobacco mutants were sensitive to humidity stress and proposed that NDH may retard the inhibition of photosynthesis by strengthening the proton gradient (ΔpH) and hence non-photochemical quenching. The involvement of NDH-dependent CEF in photosynthesis regulation in response to different environmental constraints has been widely investigated. Previous studies demonstrated that NDH-defective mutants of tobacco (*Nicotiana tabacum*) do not show any decrease in the photosynthetic activity compared to the WT under non-stress conditions (Burrows et al., 1998; Kofer et al., 1998; Shikanai et al., 1998; Horváth et al., 2000). The photosynthetic activity in the NDH-defective mutants is, however, sensitive to short-term severe stress, including strong light (Endo et al., 1999; Takabayashi et al., 2002), low moisture (Horváth et al., 2000), drought (Munne-Bosch et al., 2005), and extreme temperature (treatment at 4°C, Li et al., 2004; treatments at 4 or 42°C, Wang et al., 2006).

Various investigations reported previously that the NDH-dependent CEF is known to prevent stroma over-reduction, especially under stress conditions (Rumeau et al., 2007; Shikanai, 2007). It has been proposed that CEF is essential to balance the chloroplast energy budget (Kramer et al., 2004; Livingston et al., 2010). Livingston et al. (2010) have isolated a new class of mutant in *A. thaliana*, hcef for CEF, which shows constitutively high CEF and higher expression of NDH. Kramer et al. (2004) suggested that the regulation of CEF is essential to fulfill its proposed role in balancing the ATP/NADPH output ratio; too much activity will result in depletion of ADP, while too little will result in over-reduction of the e^-^ transport chain. Some groups have reported substantial increase in CEF under environmental stress, such as drought (Jia et al., 2008; Kohzuma et al., 2009) or high light (Baker and Ort, 1992), or during the induction of photosynthesis from prolonged dark acclimation (Joët et al., 2002; Joliot and Joliot, 2002). All these suggest a role for the NDH-dependent CEF in the regulation of the redox state of both the stroma and also the electron transfer chain, particularly under stress.

In addition to the role of NDH-dependent CEF during these stresses, recent research has also shown a crucial role of the cyclic e^-^ transport around PSI (Havaux, 1996; Schrader et al., 2004; Quiles, 2006; Zhang and Sharkey, 2009) under conditions where either the carbon metabolism or the electron transfer through the chain is retarded. During the first seconds of illumination of dark-adapted leaves, CEF operates at higher rate of about 130 s^-1^ (Joliot and Joliot, 2006); however, LEF operates at a low rate equal to 15 s^-1^ and this is owing to inactivation of the Calvin–Benson cycle in a dark-adapted leaf (Joliot and Joliot, 2006). Furthermore, in the presence of DCMU, CEF operates transiently at similarly high rate (Joliot and Joliot, 2002; Joliot et al., 2004).

Many environmental factors, such as excess or lack of light, temperature, and CO_2_ can influence PSII (Baker and Long, 1986; Baker and Bowyer, 1994). In particular, photosynthesis has been recognized as one of the most temperature-sensitive process in plants (Berry and Björkman, 1980; Quinn and Williams, 1985; Yordanov et al., 1986). For example, it has been reported previously that high temperature results in the loss of the membrane stacking due to the dissociation of the peripheral antenna complex of PSII from its core complex (Armond et al., 1980; Gounaris et al., 1984; Srivastava et al., 1997). Other studies have demonstrated that heat inactivation of chloroplasts is correlated with Mn release from the water splitting system (Nash et al., 1985; Brudvig et al., 1989), however, other researchers proposed a slowdown in the e^-^ transport from the Q_A_ to the Q_B_ in the acceptor side of PSII (Bukhov et al., 1990).

Using a rice minicore diversity panel (Li et al., 2010), our lab systematically screened the CEF around PSI and found substantial variations in the capacity of CEF between accessions. Considering that CEF can potentially protect the photosynthetic intersystem carriers from over-reduction, the aim of the present work was to investigate the degree of susceptibility of both PSII and PSI to short-term heat stress in two rice groups, i.e., one with low rate of CEF (lcef) and another with high rate of CEF (hcef). Our hypothesis was that both PSI and PSII would be more resistant to heat stress in hcef. In addition, considering that CEF includes both NDH-dependent and FQR-dependent CEF, we tested whether these two pathways could compensate for each other when the activity of one pathway is decreased under stress. Our results show that the NDH-dependent CEF may compensate the function of the FQR-dependent CEF under heat stress conditions. Our study shows the beneficial effect of moderate heat stress in promoting the NDH-pathway and how the latter (NDH) might prevent over-reduction of the stromal components in rice.

## Materials and Methods

### Plant Growth Conditions

Seeds of 12 rice accessions were germinated in petri-dishes on wet filter papers under ambient temperature conditions (25°C) in the dark until the emergence of the radicle. These 12 rice accessions were classified into two groups with different CEF capacities. The first group was characterized by a high NDH-dependent CEF around PSI, which we designated as hcef. The hcef group includes the following six accessions: Q4149, Q4143, T4172, K4099, Y4213, and G4063. The second group was characterized by a low NDH-dependent cyclic pathway which we designated as lcef and which contains the six following accessions: C4023, S4163, J4087, F4051, F4054, and P4140 (Supplementary Table 2, data of PIR at 25°C). After emergence of the radicle the plants were transferred into pots with soil (SIA Pindstrup Substrate, Rïgas Ielä, Baloži, Kekavas Novads, LV-2128, Latvia) and incubated in a growth chamber at 28/25°C (day/night) with a 12 h photoperiod and photon flux density of 600 μmol photons m^-2^ s^-1^ and 75% relative humidity. Water and nutrients were routinely added to prevent any growth limitation. Mature, healthy and non-senescent leaves from 4–5 week-old plants were used for our measurements.

### Determination of Photosystem II (PSII) Parameters

We used the Multifunctional Plant Efficiency Analyser (M-PEA; Hansatech, King Lynn, Norfolk, UK) for the estimation of the PSII parameters. Assemblies of three emitters and four detectors are built in the M-PEA sensor unit. In this instrument, wavelengths of light (from Light Emitting Diodes, LEDs) are: 625 ± 10 nm for the AL; 820 ± 25 nm for the modulated light; and 735 ± 15 nm for the FR-light. Plants were kept overnight at 25°C in darkness. Then, the detached healthy and fully expanded untreated or heat treated leaves were exposed for 0.5 s to saturating orange–red (625 nm) AL (5000 μmol m^-2^ s^-1^) provided by the LED. The ratio of variable fluorescence *F*_v_ (*F*_m_ – *F*_0_) to *F*_m_ (*F*_v_/*F*_m_) was used to evaluate the maximum efficiency of PSII. *F*_m_ (P level) represents the maximum yield of chlorophyll *a* fluorescence and *F*_0_ (0 level) is the minimum chlorophyll *a* fluorescence (the intensity of chlorophyll *a* fluorescence of dark-adapted sample with a measuring beam of negligible AL intensity). *F*_v_/*F*_0_ parameter represents the functional reaction center of PSII.

### Heat Treatment

The leaves were floated on tap water pre-warmed to respective temperatures in a controlled water-bath for 15 min. Control leaves were treated for 15 min at 25°C. After the temperature treatments in the dark, the leaves were kept at room temperature covered with moist tissue paper for the analysis of Chl fluorescence and leaf absorbance changes at 820 nm.

### Inhibitor Treatments

Whole leaves were floated in a shaker (120 rpm) on a solution containing DCMU (300 μM) alone or DCMU together with MV (200 μM) 60 min in darkness or dim light. In the latter case, MV was added 45 min after the leaves were floated in DCMU solution to avoid oxidative damage elicited by this agent. The stock solutions of DCMU were prepared in ethanol and MV in water. All inhibitor solutions were prepared fresh.

### Redox State of P_700_

Photooxidation/reduction kinetics of P_700_ was monitored in dark-adapted leaves at 25°C as the light-induced absorbance changes at 820 nm (ΔA_820_) using the ED-P_700_DW dual wavelength unit connected via a PAM-101 fluorometer (Walz). The ED-P_700_DW detects strictly the differential absorbance changes (810 – 830 nm) peaking at a single wavelength band 820 nm ascribed to the P_700_^+^ cation radical absorption and removes the plastocyanin absorbance changes (Herbert et al., 1995; Klughammer and Schreiber, 1998). The photooxidation was induced by a beam of FR-light (peaking wavelength is 735 nm; intensity of ∼14 μmol photons m^-2^ s^-1^) obtained from a light emitting diode (LED, Roithner Lasertechnik GmbH, Vienna, Austria).

### Post-illumination Rise (PIR) of Chl Fluorescence Measurement

Chl fluorescence was measured according to Schreiber et al. (1986, 1988) using a pulse-amplitude modulated fluorimeter (PAM 101, Walz). Whole rice leaves obtained from dark-adapted plants at room temperature (25°C) were used in the present experiment. The modulated non-actinic measuring beam (1.6 kHz) was switched on to obtain the dark fluorescence level or initial fluorescence (*F*_0_) where the PSII centers remain in an open state. Maximum quantum efficiency of PSII was assessed as *F*_v_/*F*_m_ to ensure that we used a healthy leaf for our experiment. Using a previously described method with slight modifications (Shikanai et al., 1998), a transient post-illumination increase in Chl fluorescence was recorded after termination of the 5 min illumination by AL (600 μmol m^-2^ s^-1^). The post-illumination rise (PIR) is explained as the re-reduction of the PQ pool in the dark by e^-^ driven by the NDH-pathway.

### Statistical Analysis

Statistical analysis was performed using ANOVA followed by pairwise comparisons of Tukey’s test, *P* < 0.05. The difference between the Q4149 and C4023 accessions was statistically significant for the initial rate (IR) at 42°C, for both PSI and PSII activities and the PIR. The differences between treatments and cultivars were assessed using two-way ANOVA with R software version 3.1.2 and results of statistical analysis are shown in Supplementary Tables 1 and 2.

## Results

### Oxidation Kinetics of P_700_ Measured with FR-Light

**FIGURE 1:**
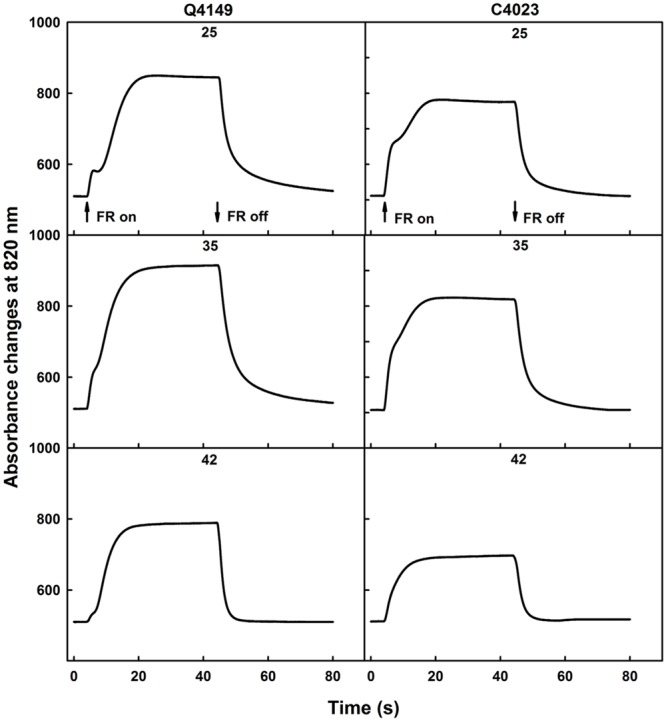
**Typical traces of 40 s FR-light induced oxidation/reduction kinetics of P_700_ monitored as absorbance changes at 820 nm (ΔA_820_) in dark-adapted leaves of Q4149 and C4023 rice accessions in untreated (25°C) control leaves **(Upper)** or exposed to 35 **(Middle)** or 42°C **(Bottom)** for 15 min.** The upward and downward arrows indicate the onset and termination of 14 μmol photons m^-2^ s^-1^ FR-light. Each curve is the average of at least six measurements.

**Figure 1** illustrates the photooxidation and the following dark re-reduction kinetics of P_700_ recorded as the absorbance changes at 820 nm (ΔA_820_) using a 40 s pulse of saturating FR (14 μmol photons m^-2^ s^-1^) in dark-adapted rice leaves (**Figure 1**). The photooxidation kinetics of P_700_ in untreated dark-adapted leaves shows a biphasic pattern for both Q4149 and C4023 rice accessions (**Figure 1**). An initial quick absorbance rise was followed by a noticeable dip for Q4149. Then, the oxidation of P_700_ proceeded slowly and reached steady-state conditions well before 40 s for both Q4149 and C4023. Under control temperature (25°C), in the case of Q4149, 25 s are required to reach the steady-state, however only 20 s are sufficient to reach this level in C4023 (**Figure 1**; upper). This means that there is more FQR-dependent CEF operating in Q4149 than in C4023. The fast oxidation of P_700_ reached the maximum rapidly during the first 200 ms for both rice accessions. According to the literature, this fast phase of P_700_ oxidation corresponds to the reduction of the stromal NADP^+^, the immediate acceptor pool of PSI (Joliot and Joliot, 2006; Govindachary et al., 2007). The amplitude of this phase is slightly higher in C4023 than in Q4149, suggesting a greater NADP^+^ pool in C4023. The slow phase of P_700_ oxidation was more delayed (retarded) in Q4149 than that in C4023 suggesting the operation of an efficient Fd-dependent FQR pathway that can compete with the LEF from Fd_red_ to NADP^+^ during the photosynthetic induction period (Joliot and Joliot, 2006).

When leaves were exposed to 35°C for 15 min and then irradiated with FR-light, we observed also a biphasic kinetics of P_700_ oxidation (**Figure 1**, middle) in which the amplitude of the fast phase representing the pool size of NADP^+^ didn’t seem to be affected following mild heat stress in both rice accessions. In addition, the slow phase of P_700_ photooxidation occurred earlier when compared to the same in untreated control leaves (**Figure 1**, compare 25–35°C for each rice accession). We ascribe the above changes to a partial inhibition of the FQR because it can still compete with the LEF. FR-light induced P_700_ oxidation attained the steady-state conditions much earlier than what was obtained for the untreated control leaves (**Figure 1**). Besides, the P_700_ oxidation was increased because of the limited restriction of the Fd-dependent pathway (FQR). When leaves were obtained from dark-adapted rice plants of both accessions (Q4149 and C4023) and then exposed to 42°C for 15 min, the P_700_ oxidation was typically rapid and monotonic, mostly for the C4023 accession, because of an early establishment of the steady-state conditions (**Figure 1**, bottom). We observed again a decline in the P_700_ oxidation amplitude compared to the control leaves (**Figure 1**). With exception for the Q4149 accession, the NADP^+^ pool was not completely depleted in dark-adapted leaves following 44°C heat treatment for 15 min. This explains that Q4149 supports more high temperature stress than C4023. In light of this result, it looks like Q4149 retains a very small amount of NADP^+^ preserving thus a very weak competition between LEF and FQR. This might explain why the Calvin–Benson cycle enzymes, especially the Rubisco activase, were not completely depressed and deactivated for this accession at this temperature and were still able to fix CO_2_ even at low rate.

Previous studies performed on *A. thaliana* confirmed the loss of the FQR component in favor of the recombination process and NDH-pathway following high temperature stress (Essemine et al., 2011). According to our above interpretation, it seems that, under high temperature stress, the re-reduction of P_700_^+^ in the dark after switching off the FR-light is monitored mainly by the recombination process and the NDH-dependent CEF since the FQR was already suppressed.

### Effect of Short-term Heat Stress on the Dark Re-reduction of P_700_^+^ after Turning off FR-Light

**FIGURE 2:**
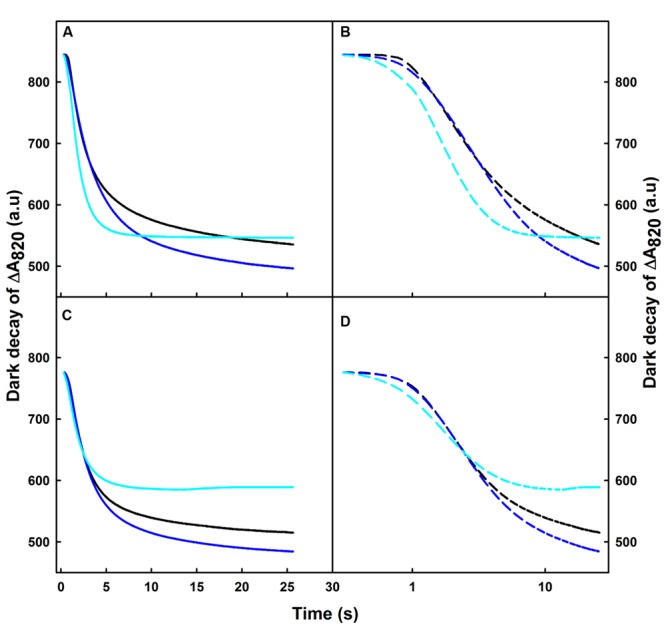
**Dark-decay of oxidized P_700_ (P_700_^+^) after switching off FR-light in untreated control (25°C, black curves) or heat treated, for 15 min to 35 (blue curves) or 42°C (cyan curves), leaves obtained from dark-adapted plants of Q4149 **(A,B)** and C4023 **(C,D)**.** Dark-decay of ΔA_820_ was plotted on a linear time scale **(A,C)** or plotted on the logarithmic scale **(B,D)**.

**FIGURE 3:**
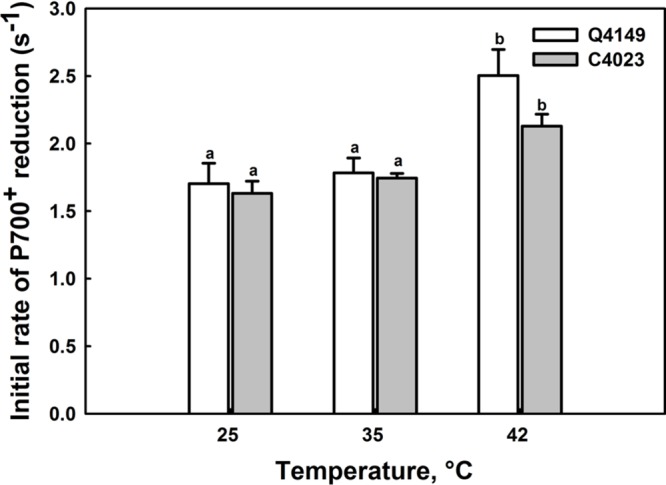
**Effects of short-term (15 min) moderate (35°C) and high (42°C) temperatures on the initial rates (IR; 0 – 1 s) of P_700_^+^ re-reduction after switching off the FR light (14 μmol m^-2^ s^-1^ during 40 s) in Q4149 and C4023.** Each data point is the average of six different measurements. The vertical bars indicated the standard errors. Letters a,b above bars indicate significant differences at *P* = 0.05 among the two rice accessions at various temperatures treatments.

After switching off the FR light, we observed a re-reduction of the oxidized P_700_ (P_700_^+^) in the dark (**Figures 1** and **2**). To better understand the effect of various temperature treatments on the amplitude of P_700_^+^ and its re-reduction in the dark, we normalized our original data of heat treated samples to the control level for both Q4149 and C4023 and plotted all on a linear scale (**Figures 2A,C**). However, to properly distinguish the effect of temperature on the IR of the re-reduction of P_700_^+^ for the same accession and clarifying the difference between accessions (Q4149 and C4023); we plotted the dark-decay curves on a logarithmic scale after normalization to the control sample (**Figures 2B,D**). In this regard, our results shown in **Figures 1** and **2** demonstrate that the dark-decay under control temperature follows the same trend and evolved in the same manner in both Q4149 and C4023 (**Figure 2**, black curves). When leaves were exposed to 35°C, the dark-decay was accelerated slightly but remained indistinguishable from the control on a linear scale in both accessions (**Figures 2A,C**). By examining **Figure 2B**, we observe that the dark decay was a little bit faster in Q4149 than C4023 (**Figure 2B**, blue curves and **Figure 3**). After increasing the temperature to 42°C, the dark decay of P_700_^+^ after turning off the FR-light was faster than under 35°C for both rice accessions (Q4149 and C4023). The re-reduction of P_700_^+^ was more significant in Q4149 than in C4023 (**Figures 2A,B**, cyan curves and **Figure 3**). These results reflect that short-term heat stress accelerates the re-reduction of oxidized P_700_ (P_700_^+^) and ultimately reveal that short-term heat stress could promote the rate of electron cyclization around PSI. Our results for this section provide further support for our hypothesis suggesting that PQ pool reduction via the NDH-mediated CEF and stromal components accumulated in the light can mitigate the photoinhibition of the PM caused by over-reduction of intersystem e^-^ carriers.

### Determination of the Initial Rate (IR) of the Dark Re-reduction of P_700_^+^

It has been previously reported that the IR of the dark re-reduction of P_700_^+^ is more directly relevant to the rate of CEF and to the electron donation to the intersystem electron transport chain by the stromal reductants (Mi et al., 1992a,b; Havaux, 1996; Wang et al., 2006). However, **Figure 3** displays an acceleration of the IR of CEF in both C4023 and Q4149 rice accessions following thermal stress. At 25°C, the IR of P_700_^+^ re-reduction after switching off the FR-light was slower in C4023 than in Q4149 by about 5% (**Figure 3**). After exposure to 35°C for 15 min, the IR was accelerated in both accessions but slightly more enhanced in Q4149 (by around 3%) than in C4023 (**Figure 3**). When exposed to 42°C, this IR of P_700_^+^ re-reduction increased more for both rice accessions but to different levels. Compared to 35°C, the increase in the IR at 42°C was about 40 and 23% for Q4149 and C4023, respectively. The IR of P_700_^+^ re-reduction remains slower in C4023 than in Q4149 after exposure for 15 min to 42°C by about 17%. Taken together, our results demonstrate a removal of the FQR-dependent CEF and an enhancement of the IR after heat stress treatment (**Figures 1** and **3**). It has been reported previously that the IR is known to be accelerated after exposure of tobacco leaves to 42°C for 6 h (Wang et al., 2006). It is most likely that the CEF driven by NDH (NDH-dependent CEF) is enhanced following thermal stress conditions since the FQR-dependent CEF is abolished.

### Effect of Short-term Heat Stress on both PSII and PSI Activities

Photosystem I activity was evaluated through the changes in the magnitude of P_700_^+^ assessed by absorbance changes at 820 nm (ΔA_820_) in the presence of inhibitors of the e^-^ transport along the PETC. Following 42°C treatment, we recorded a decline in the amplitude of P_700_^+^ (**Figure 1**, bottom). This decline in the ΔA_820_ could be explained in various ways. These are (i) the destruction of a PSI sub-population owing to damage or just (ii) a merely inactivation or over-reduction of certain PSI reaction centers (P_700_) attributed to extended electron trapping in the PSI reaction center (inactivation) or to (iii) acceptor side limitation of PSI (over-reduction) which may decrease or block e^-^ acceptance from the PSI reaction centers leading thereby to a decrease in the P_700_^+^ amplitude (**Figure 1**, bottom). To resolve this ambiguity, we measured the oxidation/reduction kinetics of P_700_ at different temperatures in absence or presence of specific inhibitors (DCMU and MV). We treated leaves with diuron (DCMU) together with MV (for details see “Materials and Methods” section) and then leaves were exposed to either moderate (35°C) or high (42°C) temperature stress prior to measure the leaf ΔA_820_.

**FIGURE 4:**
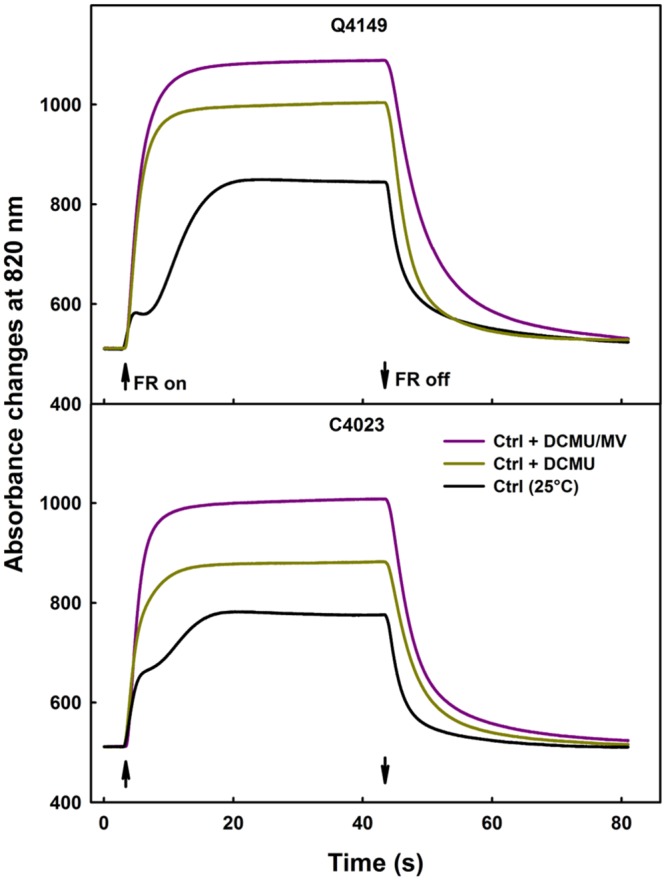
**Typical traces of P_700_ photooxidation/reduction kinetics under FR-light (14 μmol m^-2^ s^-1^) in the absence (black curves) or presence of only 300 μM DCMU (yellow curves) or together 300 μM DCMU plus 200 μM MV (pink curves) in dark-adapted leaves of Q4149 and C4023 rice accessions.** The upward and downward arrows indicate the onset and termination of 14 μmol photons m^-2^ s^-1^ FR-light. Each curve represents the average of six measurements.

**Figure 4** illustrates the data for leaves obtained from both Q4149 and C4023 accessions and exposed to control temperature (25°C). Under FR-light excitation, DCMU treated samples demonstrated not only a faster rise of ΔA_820_ but also a monotonic increase of ΔA_820_ because of an early occurrence of the steady-state condition of P_700_ photooxidation (**Figure 4**). We mentioned as well an increase in the P_700_^+^ amplitude in the presence of DCMU because this inhibitor prevents electrons from reaching P_700_^+^ from PSII via the linear route. Leaves treated with DCMU together with MV enhanced further the amplitude of P_700_^+^ oxidation by about 9 and 14% for Q4149 and C4023, respectively, compared to leaves treated only with DCMU (**Figure 4**). In addition to that, application of DCMU plus MV further delayed the P_700_^+^ re-reduction than the application of DCMU alone because DCMU weaken or blocks, depending on the concentration used, e^-^ flow between Q_A_ and Q_B_ of PSII. MV competes with ferredoxin (Kobayashi and Heber, 1994; Govindachary et al., 2007) for electrons uptake and prevents charge recombination on one hand and Fd-mediated e^-^ flow to P_700_^+^ through the Cytb_6_/f on the other.

**FIGURE 5:**
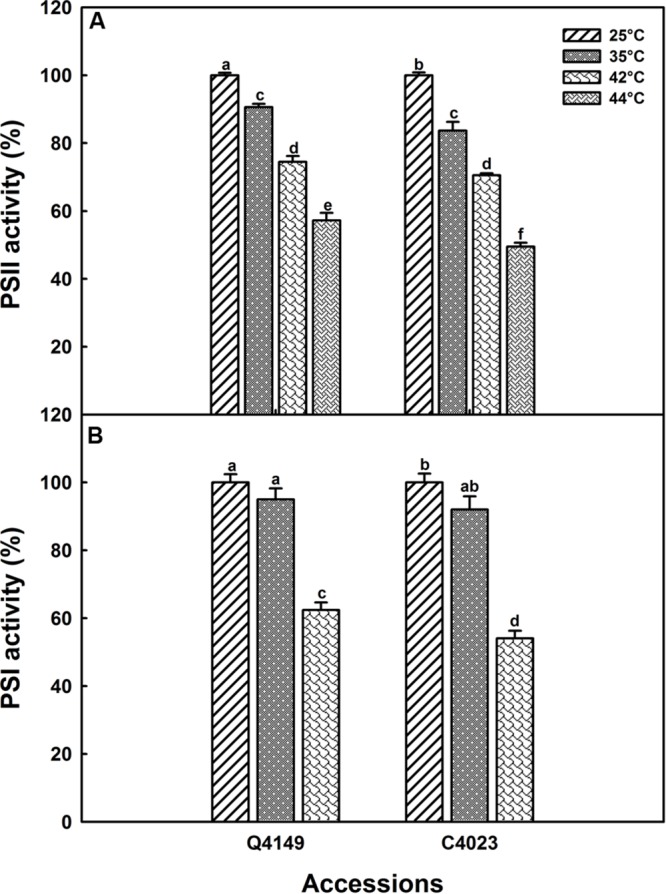
**PSII **(A)** and PSI **(B)** activities in Q4149 and C4023 leaves either untreated (Ctrl, 25°C) or exposed to 35 or 42°C for 15 min.** The PSII activity was obtained from the *F*_v_/*F*_0_ values. The amplitude of ΔA_820_ is used to express the PSI activity in leaves treated with DCMU (0.3 mM) and MV (0.2 mM) before heat exposure as shown in **Figure 4**. Each data point is the average of 10 measurements for *F*_v_/*F*_0_ and six measurements for ΔA_820_. Letters a–f above bars indicate significant differences at *P* = 0.05 among the two rice accessions at various temperatures treatments.

The same study was performed for moderate (35°C) and high (42°C) temperature stress (data not shown) to evaluate the effect of heat stress on PSI activity; results are summarized in **Figure 5**. According to our findings, no significant decrease in the PSI activity was recorded after exposure to 35°C for 15 min. However, the drop in PSI activity was about 37 and 46% in Q4149 and C4023, respectively, following treatment with 42°C for the same duration (15 min). The upper panel of **Figure 5** shows the PSII activity in both Q4149 and C4023 which is expressed as the ratio of *F*_v_/*F*_0_. Summarized results in **Figure 5** shows that after 15 min exposure of Q4149 leaves to 35, 42, or 44°C about 90, 75, and 58%, respectively, of the total fraction of PSII reaction centers are still functionally active (**Figure 5**, upper). However, after exposure of C4023 leaves to the same temperatures, the percentage of PSII reaction centers remaining active is lower and represents about 84, 71, and 50% for 35, 42, or 44°C, respectively (**Figure 5**, upper). This reveals that PSI is more susceptible to short-term heat stress than PSII in rice and the effect was more pronounced for C4023 than for Q4149. It should be noted that a 42°C treatment is sufficient to inhibit the PSI activity to approximately the same extent as for PSII activity when exposed to 44°C. Indeed, the decrease in PSII activity following treatment to 44°C was 50 and 42% for C4023 and Q4149, respectively, whereas the loss in the PSI activity after exposure to just 42°C was around 46 and 37% for C4023 and Q4149, respectively.

### Changes of PSI and PSII Activities in lcef and hcef Groups under Heat Stress

**FIGURE 6:**
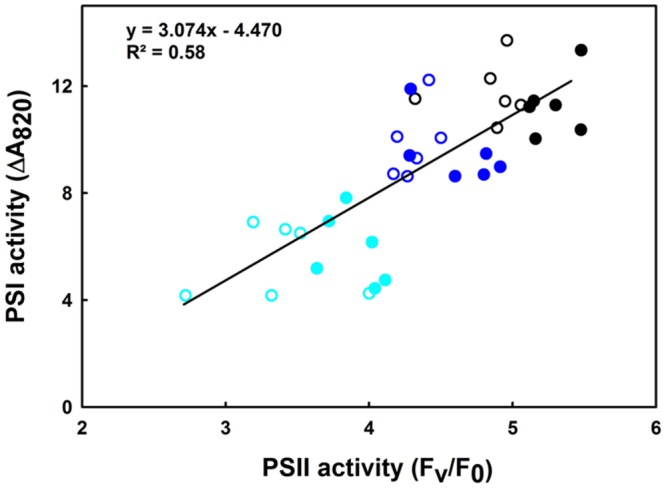
**Correlation between PSI and PSII remaining activities in leaves of 6 hcef (closed symbols) and 6 lcef (open symbols) rice accessions.** Rice leaves were exposed to 25 (black symbols), 35 (blue symbols), or 42°C (cyan symbols) for 15 min. *F*_v_/*F*_0_ (average of 10 measurements) is used as the expression of PSII activity and the amplitude of ΔA_820_ (average of six measurements) obtained in leaves treated with diuron together with MV represents the PSI activity.

This section shows the influence of moderate (35°C) and high (42°C) heat stress on the activities of PSI and PSII in both the lcef and the hcef groups. Our results show a gradually decline in the activities of both PSI and PSII (**Figures 5** and **6**). We observed a parallel loss in the primary photochemistry of PSII and the amount of active P_700_ for both the lcef and hcef groups (Supplementary Tables 1 and 2, **Figure 6**). Thus, the data summarized in Supplementary Tables 1 and 2 demonstrate a progressive decrease in *F*_v_/*F*_m_, *F*_v_/*F*_0_, and PSI activity following exposure to heat stress in all rice accessions but to different degrees between the lcef and hcef groups (Supplementary Tables 1 and 2). The decline in these parameters was more pronounced in the lcef group than in the hcef group (Supplementary Tables 1 and 2). Considering the temperature effect, the PSI and PSII activities plotted against each other for both the lcef and the hcef groups (**Figure 6**) reflect an apparent coordination in the activities between PSI and PSII. The strong positive correlation (*R*^2^ = 0.58) between PSI and PSII activities in six lcef (open symbols) and six hcef (closed symbols) has confirmed that heat treatment up to 42°C does not disrupt the PETC at all (**Figure 6**). Under heat stress, despite the damage in some PSI and PSII active reaction centers in either the lcef or hcef group, we still observe strong coordination between the PSI and PSII activities. This means that temperature 42°C doesn’t constitute a lethal or critical threshold for these two groups. As shown in **Figure 6**, this coordination is significant at ambient temperature, 25°C (**Figure 6**, black points group) and weak at high temperature, 42°C (**Figure 6**, cyan points group). Thus, it is evident that the redox poise between PSI and PSII can be more affected at high temperatures for all rice accessions studied here (**Figure 6**). However, data recorded in **Figure 6** are consistent with those of **Figure 5**. On the one hand, we obtained the highest decline in PSI activity for C4023 at high temperature, 42°C (**Figure 5**). On the other hand, the lowest PSI activity, at high temperature (42°C), was recorded for the lcef group (**Figure 6**, open cyan symbols).

### Effect of Thermal Stress on the Chl Fluorescence Evolution

The induction and relaxation of the Chl fluorescence signal following the onset of AL had similar kinetics and amplitudes in both Q4149 and C4023 when control samples (25°C) were compared to leaves getting a 15 min 35°C treatment (Supplementary Figure 1). After treatment at 42°C, we observed a reduction in the amplitude of Chl fluorescence and its faster relaxation to nearly *F*_0_ level. The heat effect was more evident in C4023 than in Q4149 when comparing the results obtained at 42°C to those recorded at 25°C (Supplementary Figure 1). We attribute the decrease in the amplitude of Chl fluorescence at 42°C under AL regime (light intensity of rice growth room) to a decreased rate of the re-reduction of the PQ pool and the faster relaxation of the Chl fluorescence might be ascribable to an enhancement in the PQ pool re-oxidation. All these changes in the evolution of Chl fluorescence may have consequences on the filling of the PQ pool in the dark, after turning off AL, via the NDH-dependent CEF.

### Effect of Thermal Stress on the NDH-Dependent PSI-CEF

**FIGURE 7:**
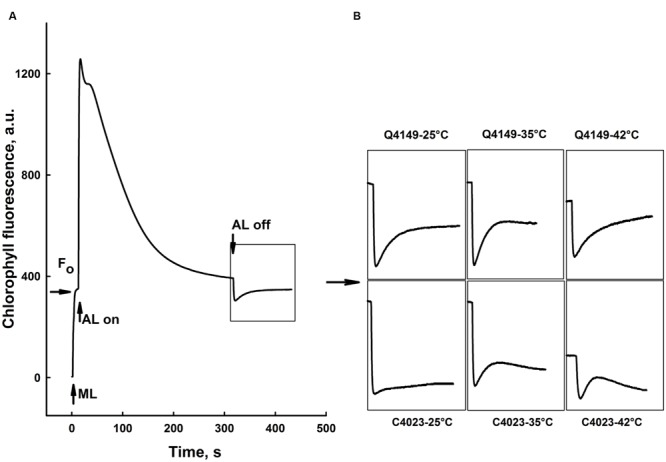
**Effects of short-term (15 min) moderate (35°C) and high (42°C) heat stress on the post-illumination rise (PIR) of Chl fluorescence in dark-adapted leaves of Q4149 and C4023 rice accessions. (A)** Chl fluorescence measured in dark-adapted untreated (Ctrl, 25°C) Q4149 leaves. *F*_0_; dark fluorescence level obtained following weak ML of 1.6 (kHz) of red light. AL, White AL (600 μmol photons m^-2^ s^-1^, lasted for 5 min). Whole leaves were obtained from dark-adapted plants and heat treated to 25, 35, or 42°C as described above and then Chl fluorescence was measured at room temperature (25°C). **(B)** Insets indicate transient rise in Chl fluorescence following light to dark transition for both Q4149 and C4023 after exposure to 25, 35, or 42°C for 15 min.

**FIGURE 8:**
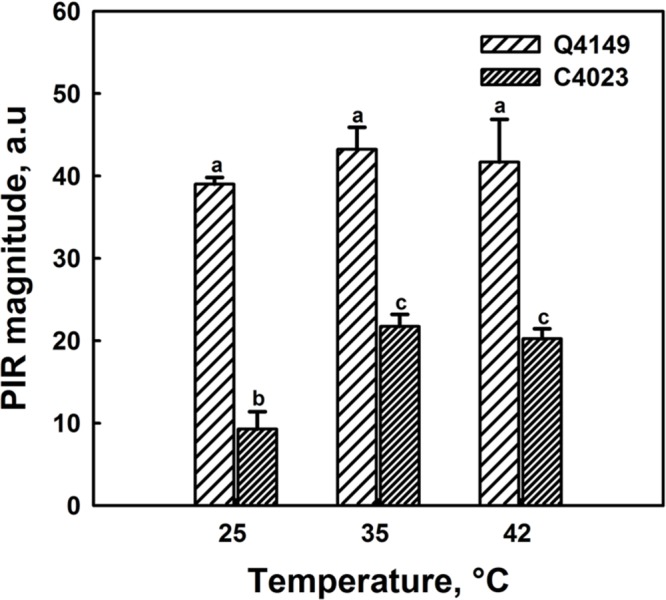
**Post-illumination rise in control (25°C) and heat treated leaves of both Q4149 and C4023 to 35 or 42°C for 15 min.** Results were expressed as the magnitude of the PIR. Data are the mean of six different measurements. The vertical bars represent the standard errors. Letters a–c above bars indicate significant differences at *P* = 0.05 among the two rice accessions at various temperatures treatments.

**Figure 7A** represents the Chl fluorescence measurement in dark-adapted leaves of Q4149 under control temperature (25°C). We assessed the *F*_0_ level by a weak measuring red light at 1.6 kHz (**Figure 7A**), after that we applied an AL of around 600 μmol photons m^-2^ s^-1^ for 5 min. Insets shown in **Figure 7B** depict the transient PIR in Chl fluorescence after switching off AL in dark-adapted leaves of both Q4149 and C4023 rice accessions (**Figure 7B**). The PIR is known to arise as a result of the reduction of the PQ pool by NAD(P)H or other reducing components that can be accumulated in the light. This reaction mainly involves the PSI-CEF driven by NDH in cyanobacteria (Mi et al., 1995) and in higher plants (Burrows et al., 1998; Kofer et al., 1998; Shikanai et al., 1998; Wang et al., 2006). Insets in **Figure 7B** demonstrate the presence of an apparent post-illumination increase in Chl fluorescence in Q4149 but it is almost absent in C4023 (very weak activity) leaves at control temperature (25°C). In leaves treated to 35°C for 15 min, we observe a slight further increase in the PIR amplitude for Q4149, however, this temperature (35°C) has a stronger effect on PIR amplitude in C4023 than in Q4149 (**Figures 7B** and **8**). In addition, when leaves where treated at 35°C, either the IR of the increasing phase or the amplitude of the PIR was enhanced in Q4149 and both undergo a slowdown (down-regulated) in this same accession (Q4149) after exposure to 42°C (**Figures 7B** and **8**). In C4023, the amplitude of PIR evolved in the same manner as in Q4149 leaves treated to 35 or 42°C (**Figures 7B** and **8**). In contrast to Q4149, the IR of the PIR in C4023 displayed a steady increase irrespective of the temperature treatment (**Figure 7B**). The increase of the PIR amplitude in the 35°C treatment was about 2.3 times (130%) compared to the control in C4023 accession (**Figure 8**); however, this enhancement is only 1.1-fold (10%) in Q4149 leaves exposed to 35°C (**Figure 8**). After the 15 min 42°C treatment of the leaves we observed a weak decrease in the PIR amplitude compared to that recorded during treatment to 35°C in both rice accessions (**Figures 7B** and **8**). It was mentioned above that the drop in the amplitude of Chl fluorescence after an application of AL under high temperature (42°C) reveals a decrease in the PQ pool re-reduction and an acceleration of its re-oxidation (Supplementary Figure 1). A possible physiological explanation for the quenching in the Chl fluorescence is a further accumulation of the reducing power in the stroma and ultimately the increase of the PIR amplitude. The discrepancy between this interpretation and our findings at 42°C suggests that the decrease in the PIR at 42°C is most likely related in part to the suppression of the FQR-dependent CEF and also to the loss of activity of some PETC super-complexes (PSI and PSII). This suggests that the FQR-dependent CEF identified as an AA-sensitive pathway (Cleland and Bendall, 1992; Joët et al., 2001) may participate in the PQ pool reduction even at a weak rate since both pathways (NDH and FQR) share a considerable part of the e^-^ flow route toward the PQ pool. Hence, the CEF competes with the LEF from Fd_red_ to NADP^+^ since both are mediated by the FNR enzyme on this part of the PETC, especially at moderate heat stress. In this regard, Endo and Asada (2002) reported that in the NDH-pathway e^-^ photoproduced at the PSI flow from NADPH to PQ pool through FNR, Fd, and NDH. In addition, Endo et al. (1998) proposed that Fd is required for the activity of the NDH pathway. These authors suggested that either NDH has the activity of FQR or NDH is FQR itself (Endo et al., 1998; Miyake, 2010).

### Thermal Stress Promotes NDH-Dependent CEF Activity in lcef Accessions

Thermal stress may promote the NDH-dependent CEF for, at least, two reasons. Firstly, heat stress leads to the obstruction of the FQR-dependent CEF in rice. In this context, our results show that FQR-dependent CEF is partially removed under moderate heat stress 35°C and it is completely abolished following high heat stress (**Figure 1**). Secondly, it has been reported in the literature that heat stress inhibits the CO_2_ assimilation leading thus to over-reduction of the stroma (Yamori et al., 2005, 2006, 2008, 2010a,b). As inhibition of CO_2_ assimilation generated by strong light, heat, chilling or water stress could lead to over-reduction of the PETC, the NDH-dependent CEF has been proposed to prevent over-reduction of the stroma, especially under stress conditions (Rumeau et al., 2007; Shikanai, 2007; Yamori et al., 2011). Therefore, it is expected to observe an enhancement in the NDH-dependent CEF, since the FQR-dependent CEF is already abolished. According to our study, the NDH-dependent CEF is improved following thermal stress conditions and this is very likely to compensate for the shortage in the FQR-dependent CEF and subsequently helps to overcome and avoid any electron pressure on the intersystem electrons carriers. To prevent over-reduction of the stromal components and avoid formation of ROS, excess of electrons photoproduced at the acceptor side of PSI must be efficiently consumed either by the Calvin–Benson cycle or by other electron valves. When CO_2_ assimilation is inhibited under heat stress (Wang et al., 2006) and the FQR-dependent CEF is abolished (**Figure 1**), alternative electron valves such as Mehler reaction and chlororespiration (**Figures 3**, **7**, and **8**) might become compulsory. In higher plants, the FQR-dependent CEF constitutes the main route of CEF around PSI, while the NDH-mediated pathway may play a compensatory function (Munekage et al., 2002, 2004; Peltier and Cournac, 2002). According to our present analysis, it is likely that the NDH-dependent CEF may constitute a second layer of plant defense and protection against harmful effects generated following exposure to severe environmental factors. In addition, the NDH-dependent CEF acts as a safety valve that protects the PM from photodamage which might be caused by an over-reduction of the e^-^ acceptors (Tolleter et al., 2011; Baltz et al., 2014).

### Modulation of the Photosynthetic Parameters of lcef and hcef Groups in Response to Heat Stress

Photosynthetic traits were similar within the two groups, lcef and hcef, and thus to avoid repetition, we have shown results only for two rice accessions (C4023 and Q4149), one from each group. We have summarized some photosynthetic parameters in Supplementary Tables 1 and 2 regarding the 12 rice accessions mentioned above. In general, irrespective of the temperature treatments, *F*_v_/*F*_m_, *F*_v_/*F*_0_, IR, PSI activity, and PIR were higher in the hcef than in the lcef group (Supplementary Tables 1 and 2). Following moderate heat stress (35°C), the PIR enhancement is more significant and surprising in the lcef group than in the hcef one. However, after exposure to high heat stress the PIR amplitude is similar to that of the untreated control sample (25°C) in the hcef but this amplitude remained too high in the lcef group compared to the corresponding control (Supplementary Table 2).

**FIGURE 9:**
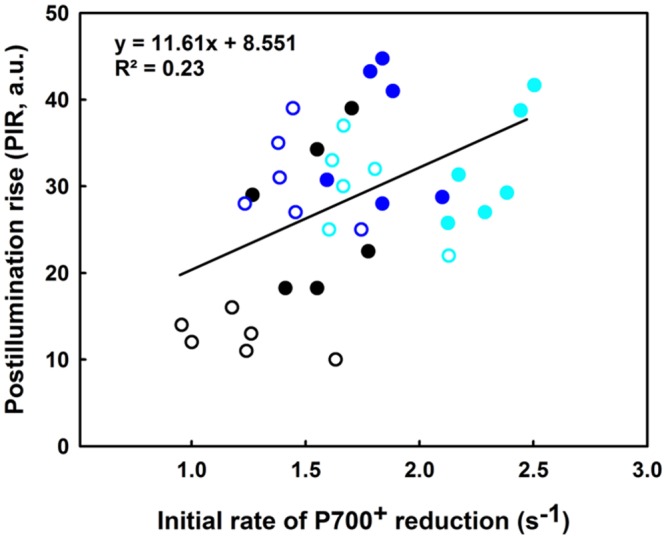
**Correlation between the initial rate (IR) of P_700_^+^ reduction (s^-1^) and the PIR in leaves of 6 hcef (closed symbols) and 6 lcef (open symbols).** Rice leaves were exposed to 25 (black symbols), 35 (blue symbols), or 42°C (cyan symbols) for 15 min. Each data point of either IR or PIR is the average of six measurements.

In regards to **Figure 9**, the slight increase in the IR is concomitant with a significant increase in the PIR for the lcef group following the 35°C treatment (compare blue and black open symbols). The reverse was recorded for hcef where we observed a lesser increase of the PIR amplitude after 35°C treatment (compared to the blue and black closed symbols). After the 42°C treatment we noted a decline in the PIR for both lcef and hcef groups (open and closed cyan symbols). With exceptions, we noticed a considerable increase in the IR for the hcef group (closed cyan symbols). The IR and PIR were used for probing two different sites of the PETC (PSI re-reduction and NDH activity) using two different techniques (ΔA_820_ for IR and Chl fluorescence for PIR). A small positive correlation between IR and PIR (*R*^2^ = 0.23) when the lcef and the hcef groups endure different temperature treatments was thus expected.

## Discussion

This study shows that there are natural variations of CEF capacities between rice lines studied here; furthermore, PSI and PSII activities in those lines with higher CEF (hcef) are more tolerant to heat stress. In addition, we show that the NDH-dependent CEF may compensate for the shortage or lack in the FQR-dependent CEF under heat stress. During this study, we used rice lines of two categories, i.e., lcef lines and hcef lines. The evidences that we can provide to confirm the segregation and classification of the 12 rice accessions into hcef and lcef are as follows: the primary photochemistry of PSII (Supplementary Table 1), FQR- and NDH-dependent CEF, PSI, and PSII activities, regardless the temperature treatment, are significantly higher in the hcef than in lcef. The CEF seems to be more operative in the hcef group than in the lcef one.

Our results show a significant difference in the photooxidation kinetics of P_700_ in dark-adapted leaves of Q4149 and C4023 under normal conditions of temperature (**Figure 1**). Furthermore, there is a partial removal of the Fd-mediated CEF, i.e., FQR, following moderate heat stress and its suppression upon severe heat stress in both lines (**Figures 1** and **2**). In line with our finding, Essemine et al. (2011) working on *A. thaliana*, found a decline in the magnitude of the middle phase mediated by Fd-dependent CEF and an increase in the fast and slow phases related, respectively, to the recombination process and chlororespiration during the reduction of P_700_^+^ following moderate heat stress. Another recent study carried out on tobacco (*Nicotiana tabacum*) demonstrated that high heat stress accelerates the IR of P_700_^+^ reduction (Wang et al., 2006) and enhances the NDH activity in WT tobacco leaves but not for the ΔndhCKJ mutant.

The influence of mild (35°C) and strong (42°C) heat stress on the activities of both PSI and PSII was analyzed in this study. Our results show that lines with low CEF, e.g., C4023, are more sensitive than lines with high CEF, e.g., Q4149, at both temperatures in both PSI and PSII activities (**Figures 5** and **6**). Furthermore, our results show that PSI is more vulnerable and susceptible to heat stress than PSII (**Figure 5**). Under heat stress, the PSI activity was dramatically suppressed (**Figure 5** and Supplementary Table 1) and photosynthetic O_2_ evolution was impaired as illustrated by the severe impediment in the structural and functional integrity of the OEC of PSII (data not shown). These results agree with a previous study on tobacco (Wang et al., 2006) showing the linear electron transport rate (ETRII) gradually slowed during 42°C stress. Under heat, the gradual decrease in the LEF (**Figures 5** and **6** and Supplementary Table 1) may progressively lead to a limitation in the CO_2_ assimilation, which may enhances the electron flow to O_2_ through the Mehler reaction, generating thus more superoxide anion radical (Asada, 1992). This is reflected in the decrease in the activities of PSI and PSII (**Figures 5** and **6**). In addition to this, operation and turnover of Rubisco activase enzymes are sensitive to high and low temperature (Kingston-Smith et al., 1997; Allen and Ort, 2001; Salvucci and Crafts-Brandner, 2004a,b), and this might further lead to stromal over-reduction. Different capacities of the NDH-dependent CEF can have differential capacity to alleviate the stromal over-reduction by channeling excess e^-^ from NAD(P)H through the NDH-dependent CEF and hence protecting photosystem from potential photooxidative damage. Endo et al. (1999) showed that repeated application of saturating light pulses resulted in a more severe photoinhibition and even chlorosis in NDH-defective mutant, while the WT sustained less photodamage and was able to recover from it.

Another key aspect of this work is that the activation of the NDH-dependent CEF compensates for a shortage in the FQR-dependent CEF. Under control temperature (25°C), C4023 showed a very small (weak) NDH activity compared to the Q4149 accession (**Figures 7B** and **8**). Following moderate heat stress, the activation of NDH was more significant in C4023 accession (lcef) than in Q4149 (hcef). This improvement in the NDH-dependent CEF might be explained as a compensatory mechanism in response to the decline in the FQR-dependent CEF under heat stress conditions. Since the FQR is abolished under heat stress in both groups, it seems that the lcef group requires more chloroplastic NDH activity to meet (or fulfill) its needs in being able to alleviate the oxidative damage caused by heat stress and for maintaining the correct ratio of ATP/NADPH production. Our results agree with this interpretation and show that, following moderate heat stress, the NDH activity rises by about 130 and 10% in C4023 (lcef) and Q4149 (hcef), respectively (**Figures 7B** and **8**). These results suggest that cyclic photophosphorylation via NDH pathway might optimize CO_2_ assimilation under heat stress, leading thus to a reduction in the generation of ROS which would inactivate CO_2_ assimilation enzymes (Ishida et al., 1998, 1999). The NDH-mediated CEF may also directly balance ATP and NADPH production, as chloroplast NDH is probably a proton pump, similar to bacterial and mitochondrial complex I. Yamori et al. (2011) reported the importance of the NDH-dependent CEF during CO_2_ assimilation and plant growth at low temperature in rice. Besides, these authors (Yamori et al., 2011) demonstrated that the lack of the NDH-dependent CEF in *crr6* mutants decreased photosynthesis (ETRI, ETRII, and CO_2_ assimilation) at both low growth temperature and low leaf temperature compared with control plants and this effect on photosynthesis caused a corresponding reduction in plant biomass.

The result that the NDH-dependent CEF can compensate for the activity of the FQR-dependent CEF in rice under heat stress is consistent with many earlier reports. Using *Arabidopsis* mutants lacking NDH and/or FQR pathway(s), Munekage et al. (2002, 2004) proved that both pathways function in a compensatory manner to prevent stroma over-reduction and to maintain an efficient photosynthesis and optimal plant growth under unstressed conditions. The *Arabidopsis* mutants defective in NDH and FQR used by Munekage et al. (2002, 2004) are *crr2-2* and *pgr5*, respectively. However, the double mutants *crr2-2pgr5* lacking both the NDH and FQR pathways could not grow photosynthetically, showing that CEF is essential for photosynthesis and that these alternative pathways could function in a compensatory way to ensure plant protection and optimize its growth (Munekage et al., 2004). Under unstressed conditions, the electrons flow rate in the FQR-dependent CEF seems much larger than that in the NDH-dependent CEF in C_3_ plants, including tobacco and *Arabidopsis* (Endo et al., 1998, 2008; Shikanai et al., 1998, 1999; Shikanai, 2007). Similarly, it has been documented previously that the NDH complex of plant thylakoid membrane is present in very low amounts, i.e., estimated to 0.2% of the thylakoid protein (Sazanov et al., 1998). On the other hand, Martín et al. (1996) demonstrated that barley (*Hordeum vul*gare) leaves incubated under photooxidative conditions exhibit a large increase in the NdhA sub-unit, suggesting that the NDH might be involved in chloroplasts protection against photooxidative stress. The NDH activity and NdhK in chloroplasts also increase after exposure of tobacco (*Nicotiana tabacum*) plants to high heat stress of 50°C in the light (Yao et al., 2001).

Under heat stress, the increase of the NDH-dependent CEF may not only alleviate the over-reduction of the stroma, but also mitigate the over-reduction of the intersystem e^-^ carriers. It seems likely that NDH strengthens proton gradients, thereby promoting the xanthophyll cycle and alleviating stroma over-reduction (Li et al., 2004). Horváth et al. (2000) have shown that ndhB-deficient tobacco mutants were sensitive to moisture stress and proposed that NDH may retard the inhibition of photosynthesis by strengthening proton gradients (ΔpH). A recently published work by Shikanai (2014) reported that in the absence of the PGR5 protein, chloroplast NDH compensates for the reduced ΔpH formation to some extent. In addition to this role of alleviating stroma over-reduction by NDH under stress conditions, the rate of e^-^ donation to NADP^+^ can also be limited by the reverse reaction of Fd-NADP^+^ reductase (FNR) which is essential for providing e^-^ to Fd in the dark (Shikanai, 2007), which can happen under high heat stress.

Nakamura et al. (2013) showed that during the evolution of NADP-malate enzyme type C_4_ photosynthesis, the proteins related to cyclic electron transfer around PSI, such as NDH-H, a subunit of NADH dehydrogenase-like complex, were promoted. Earlier work has shown that the evolutionary selection for C_4_ photosynthesis occurs in both highly associated with open and arid environments, which usually has high temperatures (Sage, 2004; Osborne and Freckleton, 2009). It is hence likely that the increased abundance of the NDH-dependent CEF in the ancestors of C_4_ plants might be involved in coping with heat stress, in addition to the commonly assumed role of the cyclic electron transfer in generating more ATP needed for C_4_ photosynthesis.

In summary, two key findings can be deduced from the present study. Firstly, the NDH-dependent CEF represents a second layer of plant defense in the absence of the FQR-dependent CEF in the lcef and hcef rice groups. Secondly, in response to heat stress, the NDH-dependent CEF was more activated in the lcef than in the hcef accessions, which may functionally protect the lcef group under conditions where FQR-dependent CEF is decreased. The physiological relevance of the activation of the NDH-dependent CEF *in vivo* during environmental stress is manifold. In particular, the onset of the cyclic process triggers the generation of extra ATP that ensures the energetic requirements for acclimation and recovery processes. It helps alleviate the over-reduction of the stroma. Furthermore, it also provides the development of a large proton gradient in order to down-regulate PSII and improve non-photochemical energy dissipation, which is a prerequisite for the plants to cope with dynamic changes in the environment.

## Author Contributions

X-GZ and JE conceived the experiment. JE and MQ conducted the experiment. JE and X-GZ wrote the paper. JE, X-GZ, and HM revised the manuscript. All authors proofed the final manuscript.

## Conflict of Interest Statement

The authors declare that the research was conducted in the absence of any commercial or financial relationships that could be construed as a potential conflict of interest.

## Abbreviations

AA: antimycin A
ADP: adenosine diphosphate
AL: actinic light
ATP: adenosine triphosphate
CEF: cyclic electron flow
Chl: chlorophyll
*crr2-2*: chloro-respiratory reduction
Cytb_6_/f: cytochrome b_6_/f
DCMU: 3-(3,4-dichlorophenyl)-1,1-dimethylurea
e^-^: electron
Fd: ferredoxin
FNR: ferredoxin-NADP^+^ oxidoreductase
FQR: ferredoxin-quinone oxidoreductase
FR-light: far-red light
hcef: high cyclic electron flow
LEF: linear electron flow
lcef: low cyclic electron flow
LHCII and LHCI: light harvesting complexes of PSII and PSI, respectively
ML: measuring light
MV: methyl viologen
NDH: chloroplast NAD(P)H dehydrogenase
OEC: Oxygen-evolving complex
P_700_: reaction center chlorophyll of photosystem I
PETC: photosynthetic electron transport chain
*pgr5*: proton gradient regulation
PM: photosynthetic machinery
PQ: plastoquinone
PS: photosystem
Q_A_ and Q_B_: primary and secondary electron acceptor of PSII, respectively
ROS: reactive oxygen species
Rubisco: ribulose-1,5-bisphosphate carboxylase/oxygenase
WT: wild type
