# Supplementary material for: Response of Chloroplast NAD(P)H Dehydrogenase-Mediated Cyclic Electron Flow to a Shortage or Lack in Ferredoxin-Quinone Oxidoreductase-Dependent Pathway in Rice Following Short-Term Heat Stress
Source: Front Plant Sci. 2016 Mar 30;7:383. doi: 10.3389/fpls.2016.00383 (PMC4811871; doi:10.3389/fpls.2016.00383)
Supplement: Supplementary file 1 [file Data_Sheet_1.PDF]

Supplementary Figure 1: Measurement of Chl fluorescence in dark-adapted untreated (25 ℃) or heat-treated leaves of Q4149 and C4023 rice accessions to moderate (35 ℃) or high (42 ℃) temperature for 15 min.

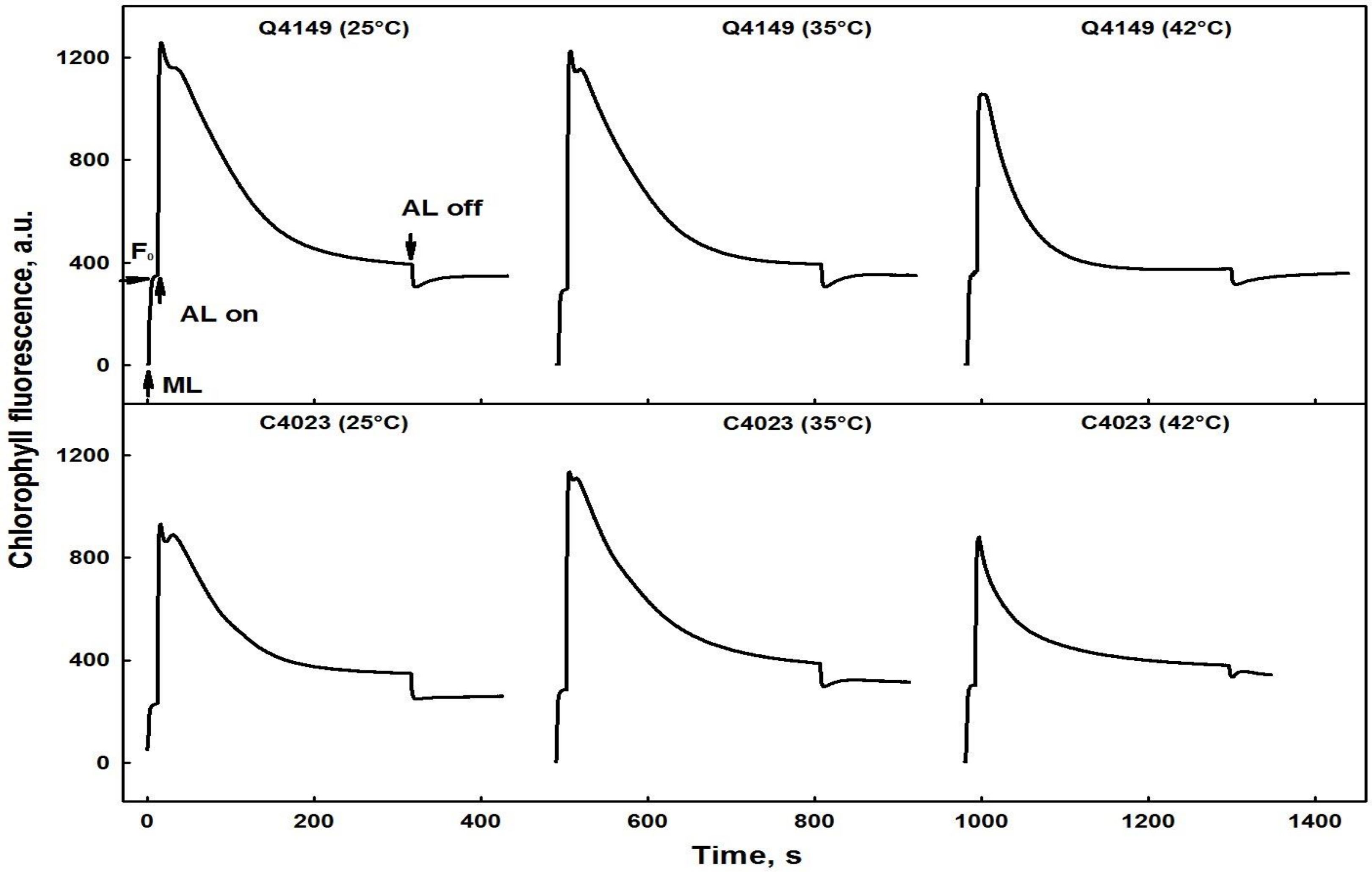

Supplementary Table 1: The maximum efficiency of PSII (F_v/F_m) and the PSII activity (F_v/F_0) in 6 lcef and 6 hcef rice accessions. Leaves were untreated (25ºC) or exposed to 35, 42 or 44ºC for 15 min. The leaves were dark-adapted on their plants before detachment and exposure to any heat treatment. Each value is the mean of 10 measurements.

**Parameters**	**F_v/F_m**				**F_v/F_0**			
**Temperatures (ºC)**	**25**	**35**	**42**	**44**	**25**	**35**	**42**	**44**
**lcef rice accessions**								
C4023	0.83 ±0.001	0.81 ±0.002	0.77 ±0.004	0.72 ±0.005	4.85 ±0.035	4.20 ±0.046	3.42 ±0.083	2.53 ±0.06
S4163	0.83 ±0.001	0.82 ±0.001	0.76 ±0.006	0.75 ±0.006	4.89 ±0.048	4.33 ±0.24	3.19 ±0.065	3.13 ±0.096
J4087	0.81 ±0.002	0.81 ±0.002	0.73 ±0.005	0.67 ±0.017	4.32 ±0.045	4.17 ±0.05	2.72 ±0.063	2.06 ±0.142
F4051	0.83 ±0.002	0.83 ±0.004	0.80 ±0.003	0.69 ±0.011	4.95 ±0.06	4.50 ±0.118	4.0 ±0.073	2.22 ±0.102
F4054	0.84 ±0.002	0.80 ±0.002	0.77 ±0.007	0.73 ±0.006	5.06 ±0.055	4.27 ±0.06	3.32 ±0.149	2.74 ±0.085
P4140	0.83 ±0.001	0.82 ±0.001	0.78 ±0.009	0.72 ±0.011	4.96 ±0.041	4.42 ±0.039	3.52 ±0.16	2.63 ±0.13
**hcef rice accessions**								
Q4149	0.84 ±0.001	0.81 ±0.005	0.79 ±0.001	0.73 ±0.008	5.12 ±0.044	4.28 ±0.13	3.72 ±0.03	2.77 ±0.11
Q4143	0.84 ±0.002	0.83 ±0.004	0.80 ±0.003	0.76 ±0.006	5.15 ±0.078	4.82 ±0.138	4.02 ±0.072	3.20 ±0.105
T4172	0.84 ±0.002	0.82 ±0.002	0.78 ±0.005	0.77 ±0.005	5.16 ±0.087	4.60 ±0.07	3.64 ±0.105	3.31 ±0.096
K4099	0.84 ±0.001	0.83 ±0.001	0.80 ±0.004	0.72 ±0.011	5.30 ±0.034	4.80 ±0.044	4.11 ±0.104	2.71 ±0.145
Y4213	0.85 ±0.001	0.81 ±0.003	0.805 ±0.002	0.8 ±0.006	5.48 ±0.051	4.29 ±0.08	4.15 ±0.047	3.49 ±0.123
G4063	0.85 ±0.001	0.83 ±0.002	0.80 ±0.001	0.75 ±0.004	5.48 ±0.045	4.91 ±0.085	4.04 ±0.024	2.92 ±0.052
Temperature	$3.65\ 10^{-8}$ (P<0.001)***				$4.4\ 10^{-11}$ (P<0.001)***			
Cultivar	0.477 (ns)				0.198 (ns)			
Temperature x Cultivar	0.989 (ns)				1.00 (ns)			

Supplementary Table 2: PSI activity, initial rate (IR) of the re-reduction of P_{700}^{+} (s^{-1}) and post-illumination rise (PIR) in 6 lcef and 6 hcef rice accessions. Leaves were untreated (25ºC) or heat-treated to 35 or 42ºC for 15 min. Leaves were dark-adapted on their plants before detachment and exposure to any heat treatment. Each value is the mean of 6 measurements.

**Parameters**	**IR**			**PSI activity**			**PIR**		
**Temperatures (ºC)**	**25**	**35**	**42**	**25**	**35**	**42**	**25**	**35**	**42**
**lcef rice accessions**									
C4023	1.63 ±0.09	1.74 ±0.03	2.13 ±0.09	12.28 ±0.07	10.15 ±0.20	6.64 ±0.30	10 ±2.13	25 ±1.45	22 ±2.97
S4163	1.00 ±0.06	1.23 ±0.05	1.60 ±0.09	10.44 ±0.15	9.3 ±0.18	6.91 ±0.13	12 ±1.63	28 ±1.53	25 ±1.25
J4087	1.26 ±0.03	1.39 ±0.08	1.67 ±0.06	11.52 ±0.24	8.72 ±0.08	4.17 ±0.69	13 ±1.42	31 ±2.50	30 ±1.70
F4051	0.96 ±0.02	1.38 ±0.05	1.62 ±0.05	11.43 ±0.22	10.06 ±0.49	4.25 ±0.79	14 ±0.47	35 ±2.45	33 ±2.50
F4054	1.18 ±0.02	1.44 ±0.08	1.67 ±0.07	11.29 ±0.19	8.63 ±0.07	4.16 ±0.64	16 ±2.16	39 ±1.57	37 ±3.64
P4140	1.24 ±0.02	1.46 ±0.02	1.80 ±0.04	13.70 ±0.68	12.22 ±0.30	6.5 ±0.34	11 ±1.25	27 ±3.44	32 ±1.83
**hcef rice accessions**									
Q4149	1.70 ±0.15	1.78 ±0.11	2.50 ±0.26	11.22 ±0.27	9.40 ±0.25	6.94 ±0.25	39 ±0.82	43.25 ±2.64	41.67 ±5.21
Q4143	1.27 ±0.09	1.88 ±0.09	2.17 ±0.06	11.44 ±0.13	9.48 ±0.19	6.16 ±0.18	29 ±0.82	41 ±6.19	31.33 ±7.27
T4172	1.41 ±0.12	1.6 ±0.02	2.10 ±0.14	10.03 ±0.19	8.63 ±0.27	5.18 ±0.26	18.25 ±0.73	30.75 ±1.52	25.75 ±3.42
K4099	1.55 ±0.09	1.84 ±0.08	2.44 ±0.04	11.29 ±0.18	8.687 ±0.23	4.75 ±0.38	34.25 ±1.28	44.75 ±3.42	38.75 ±4.44
Y4213	1.78 ±0.10	2.10 ±0.11	2.38 ±0.07	13.33 ±0.72	11.89 ±0.22	9.82 ±0.28	22.5 ±1.11	28.75 ±2.33	29.25 ±1.28
G4063	1.55 ±0.06	1.836 ±0.02	2.29 ±0.10	10.36 ±0.09	8.98 ±0.19	4.43 ±0.63	18.25 ±1.73	28 ±1.25	27 ±0.67
Temperature	0.23 (ns)			$3.86\ 10^{-10}$ (P<0.001)***			0.00041 (P<0.001)***		
Cultivar	0.63 (ns)			0.777 (ns)			0.0054 (P<0.01)**		
Temperature x Cultivar	0.92 (ns)			0.219 (ns)			0.05476 (P≤0.05)*		
